# Photoreceptor outer segments promote the accumulation of lipid droplets in microglia by reprogramming lipid metabolism

**DOI:** 10.3389/fimmu.2026.1892763

**Published:** 2026-09-09

**Authors:** Lingling Ge, Danyang Yu, Lingyan Yan, Yingnan Lv, Ruiyao He, Ling Yuan, Ping Duan, Juncai He

**Affiliations:** 1 Department of Ophthalmology, 920th Hospital of Joint Logistics Support Force, PLA, Kunming, Yunnan, China; 2 Department of Ophthalmology, University-Town Hospital of Chongqing Medical University, Chongqing, China; 3 Southwest Eye Hospital/Southwest Hospital, Third Military Medical University (Amy Medical University), Chongqing, China; 4 Department of Ophthalmology, The First Affiliated Hospital of Kunming Medical University, Kunming, Yunnan, China

**Keywords:** cholesterol metabolism, lipid droplets, lipid-droplet-accumulating microglia, microglia, photoreceptor outer segments, retinal degeneration

## Abstract

Retinal degeneration (RD) ultimately leads to blindness owing to progressive photoreceptor loss. Previous studies have shown that microglia play a key role in photoreceptor degeneration through multiple phenotypes. However, the determinants of their phenotypic regulation remain unclear. As microglia migrate into the subretinal space and engulf photoreceptor outer segments (POSs), we aim to investigate how POSs affect microglial activation and the relevant pathways. A classic mouse microglial cell line (BV2) was used to explore the effect of POS exposure on microglia activation. Transcriptomic and lipidomic analyses were used to investigate the mechanisms by which POS treatment influenced microglial lipid metabolism. A classic RD model induced by sodium iodate (SI) was used to confirm the microglial phenotype activated by POS *in vivo*. Using a mouse microglial cell line (BV2), we found that POS exposure induced a novel microglial state distinguished by a distinctive morphological feature, a specific transcriptional signature, and a distinct lipidomic profile compared with the LPS and untreated groups. BV2 cells with prolonged POS exposure showed significantly different lipid metabolic pathways, particularly genes associated with cholesterol metabolism. This lipidomic analysis further identified a parallel increase in free fatty acid (FFA) and cholesterol ester (CE) levels, with FFA-22:6 (DHA) and CE-22:6 being the most upregulated, a key metabolic transition that might play a crucial role in promoting lipid droplet (LD) formation in POS-treated microglia. Consistent with these findings, LD accumulation was also observed in the microglia located in the photoreceptor layer of the SI-induced retinal degeneration model. Functionally, POS-treated microglia showed the suppression of interferon-responsive transcriptional programs and anti-inflammatory effects, and exhibited impaired engulfment capabilities while maintaining normal lysosomal function. Taken together, these findings reveal a lipid-mediated mechanism that drives microglial phenotypic switching and provide a more physiologically relevant *in vivo* model to mimic microglial activation in degenerative retinas than LPS stimulation.

## Introduction

Retinal degeneration (RD) refers to a group of retinal disorders that cause blindness and is typically characterized by the progressive loss of photoreceptors (1, 2). In most RD models, causative factors such as aging or genetic defects lead to primary rod-specific damage, subsequently followed by a secondary degeneration of the cones (3). However, the mechanisms governing the loss of rods and cones remain incompletely understood, creating a gap that hinders the development of effective therapeutic strategies to prevent or slow photoreceptor degeneration in RD.

Microglia are widely considered to be central contributors to photoreceptor degeneration in models of RD (4, 5). The role of microglia in RD exhibits both protective and detrimental effects, likely attributable to multiple microglial phenotypes that vary across animal models and stages of the disease (6–15). Our prior investigation indicates that microglia are activated into disease-associated microglia (DAM) in the photoreceptor layer of RD, which promotes the clearance of dead cells and attenuates neutrophil infiltration, thereby delaying photoreceptor loss by decreasing secondary damage (13). However, the mechanism driving phenotypic changes in activated microglia during RD remains largely unexplored, impeding the development of stage-specific therapies targeting microglia.

Both rods and cones exhibit a shared cellular architecture consisting of four distinct compartments: an outer segment (OS) specifically designed for phototransduction, an inner segment (IS) that facilitates the formation of OS architecture, a cell soma containing the nucleus and essential metabolic machinery, and a synaptic terminal that transmits signaling to second-order neurons (16–18). During RD, microglia migrate to the outer nuclear layer (ONL) and the subretinal space (SRS), where they engulf photoreceptor outer segments (POSs) (9, 13, 19). Nevertheless, it is not known how POS affects the microglial phenotype and function.

During retinal degeneration, activated microglia not only engulf POSs, but also phagocytose ISs, cell somas, synaptic terminals (14) and infiltrating neutrophils (13). In contrast to the complex microenvironment and multitude of confounding processes *in vivo*, *in vitro* models provide a more controlled system for investigating the molecular mechanisms underlying interactions between microglia and POS (20). At present, most studies use LPS-stimulated BV2 cells to investigate or validate signaling pathways involved in retinal microglial activation. However, LPS is not a direct activator of microglia in models of RD. Thus, the present study sought to elucidate the roles of POS in modulating microglial activation and to compare these effects to those of LPS using the BV-2 microglial model *in vitro*. To validate the effects of POS on microglia activation and function, RNA sequencing and lipidomics were performed. Then, a mouse model of RD was established via intraperitoneal injection of sodium iodate (SI) to elucidate whether the microglial phenotype induced by POSs *in vitro* was observed in the degenerative retina *in vivo*. Collectively, these findings advance our understanding of the role of POSs in shaping microglial phenotypes and the mechanisms associated with them. Moreover, this study provides a more physiologically relevant *in vitro* model for mimicking microglial activation in mouse models of RD.

## Materials and methods

### Animals

C57BL/6 mice aged 6 to 8 weeks were obtained from ENSIWEIER Biotech Ltd, located in Chongqing, China. All mice were housed in the Center for Experimental Animals at Chongqing Medical University, China, where they were kept at room temperature of 25 °C under a 12 h light and 12 h dark cycle with ad libitum access to food and water. For euthanasia, a 30% chamber replacement rate of CO_2_ was used.

### Drug administration

To create a mouse model of RD, sodium iodate (SI; Sigma, 7681552) was injected intraperitoneally at a dosage of 30 mg/kg. Age-matched control mice received an intraperitoneal injection of PBS. Morphological experiments were carried out at 7, 14, and 21 days post-SI injection, as well as 7 days following the PBS injection.

### Photoreceptor outer segment preparation

POSs were prepared from 8-week-old C57 mice using a previously published method (21). A total of 15–20 animals were used for each preparation. In brief, the retinas from mice were placed in a tube containing 300 μl of 8% OptiPrep (Nycomed, Oslo, Norway) in Ringer’s buffer (130 mM NaCl, 3.6 mM KCl, 2.4 mM MgCl2, 1.2 mM CaCl2, 10 mM Hepes, pH 7.4, containing 0.02 mM EDTA). The solution was vortexed for 1 min and centrifuged at 200g for 1 min. The supernatant, which contained the POS, was collected carefully. The collection process was repeated by dissolving the supernatant in another 300 μl of 8% OptiPrep, vortexing, centrifuging, and collecting the supernatant six times in total. The collected POS supernatants were then combined and overlaid onto a continuous 10–30% OptiPrep gradient in Ringer’s buffer. This mixture was centrifuged for 50 min at 26,500g. Finally, the POSs were harvested, diluted three times with Ringer’s buffer, and centrifuged for 30 min at 26,500g—the pelleted material contained intact POSs.

### Cell culture

BV2 microglial cells were cultured at 37 °C in a 5% CO2 incubator, using Dulbecco’s Modified Eagle Medium (SH30023.01B, Hyclone) enriched with 10% fetal bovine serum (FBS; FB25015, Clark Bioscience). The BV2 cells were treated for 48 h with 20 μg/ml POS and 1 μg/ml LPS (from E. coli, Sigma) in DMEM + 5% FBS. The control group received only the vehicle solution (PBS).

### Immunohistochemistry and BODIPY staining

Immunofluorescence staining of frozen tissue sections was performed in accordance with the previously mentioned (22). Briefly, after fixation in 4% paraformaldehyde (PFA) at 4 °C for 15 min, the eyecups were transferred to 30% sucrose at 4 °C overnight. Retinas were subsequently embedded in an optimal cutting temperature (OCT) medium (Sakura FineTek, Torrance, CA, USA) and sectioned into 15-μm sagittal slices. Slices containing the optic nerve were selected for immunostaining and analysis. For cell preparation, BV2 cells seeded on slides were fixed with 4% PFA at room temperature for 15 min.

For immunofluorescence staining, sections were washed three times with 0.01 M PBS. They were then permeabilized and blocked with PT1, a PBS solution containing 0.1% Triton X-100 and 10% goat serum, at 37 °C for 30 min. The sections were subsequently incubated overnight at 4 °C with the primary antibodies of IBA1 (Wako, 019–19741) or Rhodopsin (Millipore, MABN15) in PT2, a solution of PBS containing 0.03% Triton X-100 and 5% donkey or goat serum. After five PBS washes, the sections were incubated with fluorophore-conjugated secondary antibodies in PT2 at 37 °C for 1 h. Following three times wash with PBS, the lipid droplet was stained with BODIPY 493/503 (1:1000, Thermo Fisher) for 15 min at room temperature. Finally, nuclei were counterstained with 4’,6-diamidino-2-phenylindole (DAPI; Sigma-Aldrich). Images of immunofluorescence staining were acquired using a confocal microscopy system (Zeiss LSM 780 and Nikon AX confocal microscope).

The analysis of microglial number and morphology was conducted following the methods described in previous studies (13, 23). ImageJ (Fiji) was used to conduct quantitative morphological analysis. Images were converted to single-channel greyscale images (8-bit). The rectangular selection tool was used to capture the single cell, and the signal intensity thresholds were individually adjusted to capture the IBA1. Measurements included cell area and perimeter (based on IBA1-positive regions) and the cell shape descriptor (circularity). The perimeter-to-area ratio was used as a measure of the cell surface roughness, or the extent of ramification. The percentage of BODIPY^+^ microglia (the number of BODIPY^+^IBA1^+^ cells/total number of IBA1^+^ cells), the number, and the size of BODIPY^+^ lipid droplets in each cell were determined blinded on images. As previously described (24), total cells (IBA1^+^) and BODIPY^+^ cells in the retina or on the cell slide were manually counted from randomly selected visual fields (maximum projection of the z-stack across the whole section). To determine the number and average size of lipid droplets, the BODIPY^+^ signal was analyzed using the ‘analyze particles’ function in ImageJ in randomly selected visual fields with the maximum projection of the z-stack across the entire section.

### Phagocytosis assay

After treating the BV2 cells with LPS or POS for 24 h, FITC-latex beads (1 µM, L4655, Sigma-Aldrich) were added to the medium. Following 24 h of incubation, the BV2 cells were washed three times with PBS to remove uningested latex beads. Finally, the ingested latex beads in each BV2 cell were analyzed to quantify phagocytic capability. This analysis involved quantifying the percentage of latex bead-positive cells (the number of latex-bead^+^IBA1^+^ cells/total number of IBA1^+^ cells) and measuring the total area of latex beads in each cell using ImageJ.

### Analysis of lysosome function

The DQ-Red-BSA assay was performed according to the published protocol (dx.doi.org/10.17504/protocols.io.j8nlk8dp6l5r/v1) as previously described (25). In summary, BV2 cells were cultured and treated with LPS or POS, as described earlier. After 48 h of treatment, DQ-Red-BSA (10 μg/ml; MCE, HY-D2449) was added to the cell culture media and incubated for 6 h at 37 °C. Cells were then fixed with 4% PFA at room temperature, and images were acquired under identical conditions for all groups. Red fluorescence resulting from lysosomal targeting of DQ-Red-BSA was determined using ImageJ. We quantified the percentage of BV2 cells positive for the DQ-Red signal (the number of DAPI-positive nuclei surrounded by the DQ-BSA signal/total number of DAPI-positive nuclei) and the area of the red fluorescent signal per cell.

### mRNA sequencing

For mRNA sequencing, the control, LPS-treated, and POS-treated groups were labeled as “Con,” “LPS,” and “POS,” respectively. Total RNA was extracted from BV2 cells using TRIzol reagent (Invitrogen, Carlsbad, CA, USA) according to the manufacturer’s instructions. RNA purity and quantification were assessed with the NanoDrop 2000 spectrophotometer (Thermo Scientific, USA). RNA integrity was evaluated using the Agilent 2100 Bioanalyzer (Agilent Technologies, Santa Clara, CA, USA). Next, the libraries were constructed using the VAHTS Universal V10 RNA-seq Library Prep Kit (Premixed Version) according to the manufacturer’s instructions. The transcriptome sequencing and analysis were conducted by OE BiotechCo., Ltd. (Shanghai, China). The libraries were sequenced on an Illumina Novaseq 6000 platform, generating 150bp paired-end reads. Gene expression levels were reported as FPKM values.

Differential expression analysis was performed using DESeq2. A threshold was set at a Q value < 0.05 and a fold change > 2 or < 0.5 to identify significantly differentially expressed genes (DEGs). To further analyze the DEGs, we performed enrichment analysis using the hypergeometric distribution, focusing on Gene Ontology (GO), Kyoto Encyclopedia of Genes and Genomes (KEGG), Reactome, and WikiPathways. The analysis was carried out using R (v3.2.0) respectively. Additionally, Gene Set Enrichment Analysis (GSEA) was performed with GSEA software. The analysis used a predefined gene set, and the genes were ranked based on the degree of differential expression between the two sample types. Subsequently, we tested whether the predefined gene set was enriched at either the top or bottom of the ranking list.

### Reverse-transcription quantitative polymerase chain reaction

The RT-qPCR analysis was performed as previously described (13, 14). Briefly, residual RNA from mRNA sequencing (1μg per 20 μl reaction) was reverse transcribed using a PrimeScript™ FAST RT reagent Kit with gDNA Eraser (Takara Bio, Kusatsu, Japan). TB Green^®^ Premix Ex Taq™ II (Takara Bio, Kusatsu, Japan) was used to perform quantitative PCR on a LightCycler 96 Instrument (Roche Diagnostics, Mannheim, Germany) according to the manufacturer’s instructions. The relative expression of the mRNAs was normalized to that of glyceraldehyde 3-phosphate dehydrogenase (GAPDH). All primers were purchased from Sangon Biotech (listed in **Supplementary Table 1**).

### Lipidomic analysis

Lipidomic analysis was conducted at Oebiotech (Shanghai, China) using AB SCIEX QTRAP 6500 plus system (RRID: SCR_021831), which is equipped with an electrospray ionization (ESI) source, as previously described (26). Briefly, the lipid was extracted from BV2 cells using 600 μL of a methanol: H_2_O (1:1, v/v) solution containing the internal standards CA-D4 and GCA-C13. Next, 600 μL of chloroform was added to the mixture, which was then sonicated in an ice-water bath. The mixture was allowed to stand at 4 °C for 30 min until the liquid separated into distinct layers. Subsequently, 600 μL of the lower liquid (chloroform layer) was transferred into a liquid chromatograph mass spectrometer (LC-MS) injection vial and evaporated to dryness under vacuum. Following this, secondary extraction was performed by adding 600 μL of a chloroform-methanol (2:1, v/v) solution to a centrifuge tube. The lipid residue was then dissolved in an LC-MS vial using 300 μL of isopropanol-methanol (1:1, v/v), to which isotope internal standards were added. This mixture was centrifuged for 10 min at 13,000 rpm and 4 °C. After centrifugation, 200 μL of the supernatant was transferred to an LC-MS injection vial with a lined tube for analysis. The remaining lipid was also dissolved in an LC-MS vial containing 300 μL of an isopropanol-methanol solution (1:1). After adding mixed-isotope internal standards, it was centrifuged again at 13,000 rpm and 4 °C for 10 min. Finally, 200 μL of the supernatant was transferred to an LC-MS injection vial for further analysis. To identify significantly different lipids, we set thresholds at p-values < 0.05 and fold changes> 1.2 or < 1/1.2.

### Statistical analysis

All data are presented as the mean ± standard deviation (SD). One-way ANOVA followed by Tukey’s multiple comparisons test or Kruskal–Wallis test and Dunn’s multiple comparisons test was used for comparisons of multiple groups. All statistical analyses were conducted using GraphPad Prism 9. P-values less than 0.05 were considered statistically significant.

## Results

### The exposure to POS induced distinct microglial structural and transcriptomic characteristics *in vitro*

**Figure 1:**
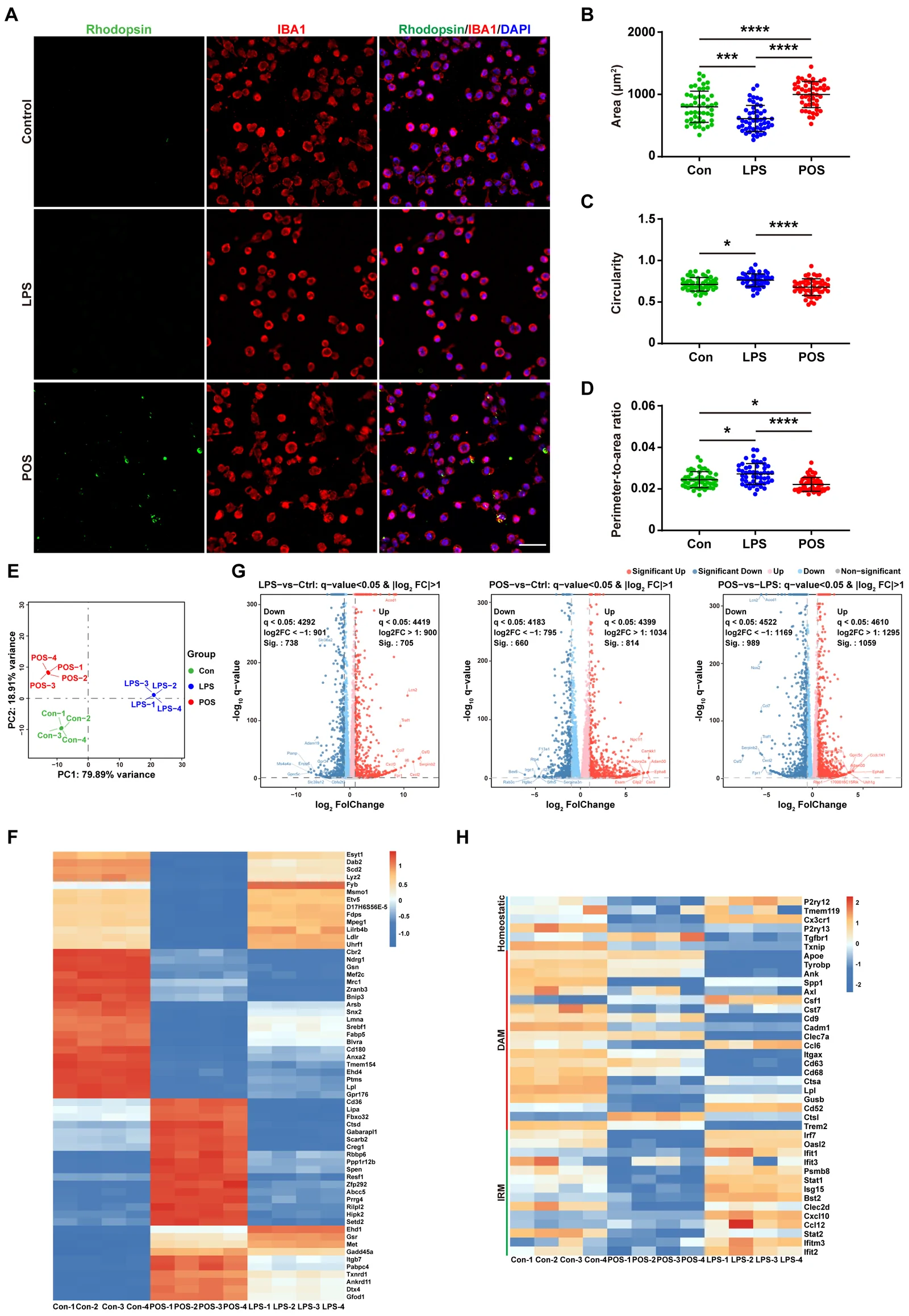
Transcriptomic and morphological alterations in LPS-treated and POS-treated microglia *in vitro*. **(A)** Representative high-resolution confocal images show immunofluorescent staining of rhodopsin, IBA1, and DAPI in BV2 cells from control, LPS-treated, and POS-treated groups. Scale bar, 50 μm. **(B–D)**. Statistical analysis of the area, circularity, and roughness in each BV2 cell from control, LPS-treated, and POS-treated groups (n = 50 cells per group). Bars represent means; error bars represent SDs. *p < 0.05, ***p < 0.001, ****p < 0.0001 using one-way ANOVA test and Tukey multiple comparisons test **(B, C)** or Kruskal-Wallis test and Dunn’s multiple comparisons test **(D)**. **(E)** The principal component analysis (PCA) plot depicts transcriptome samples from the three groups: control (green), LPS (blue), and POS (red) (n = 4 samples from distinct groups). **(F)** A hierarchically clustered heatmap highlights the top 60 most significantly differentially expressed genes following POS treatment in comparison to the control group. Color key corresponding to row Z-score. **(G)** Volcano plots show fold changes (x-axis, log2 scale) and significance (y-axis, -log10 scale) of genes in BV2 cells across the untreated group, the LPS-treated group, and the POS-treated group (n = 4 samples from distinct groups). **(H)** A heatmap presenting subsets of the genes associated with homeostatic microglia, disease-associated microglial (DAM), and type I interferon (IFN-I)-responsive microglia (IRM). Color key corresponding to row Z-score.

LPS is commonly used to investigate microglia stimulation, serving as a model to replicate the inflammatory processes associated with microglial activation (27). To investigate microglial responses to POS exposure and compare them with those to LPS, we first analyzed structural changes in BV2 cells following a 48h challenge with LPS or POS. A high-resolution confocal image showed that about 44.10% IBA1-positive microglia colocalized with rhodopsin (**Figure 1A**), suggesting that POS was internalized by BV2 cells. The morphological analysis showed that microglia treated with LPS exhibited the smallest size, highest roundness, and greatest surface roughness (**Figures 1B–D**). Conversely, POS treatment significantly increased size (**Figure 1B**) and reduced surface roughness (**Figure 1D**) in microglia compared with the untreated groups.

To examine the impact of POS and LPS on microglial transcriptomic signatures, we performed RNA-seq on BV2 cells across three groups. The first principal component separated LPS-treated microglia from the control and POS-treated groups, whereas the second component segregated POS-treated microglia from untreated controls (**Figure 1E**), suggesting marked differences between their transcriptome (**Figure 1F**). In comparison to the control group, treatment with LPS resulted in the upregulation of 705 DEGs and the downregulation of 738 DEGs (**Figure 1G**; **Supplementary Dataset 1**); conversely, the POS exposure exhibited 814 DEGs that were upregulated and 660 DEGs that were downregulated (**Figure 1G**; **Supplementary Dataset 2**). Compared with the LPS group, POS treatment elicited a transcriptomic response characterized by 1,059 upregulated and 989 downregulated DEGs (**Figure 1G**; **Supplementary Dataset 3**). As recent studies have highlighted the important roles of disease-associated microglia (DAM) and type I interferon (IFN-I)-responsive microglia (IRM) in the progression of retina diseases (13, 15, 22), we examined how LPS or POS affected these microglial phenotypes. By focusing on marker genes of DAM and IRM in mRNA sequencing analysis, the heatmap showed that both LPS and POS treatments downregulated some DAM genes (**Figure 1H**). Notably, POS treatment inhibited the expression of most IRM genes compared with both the LPS-treated and untreated groups (**Figure 1H**).

To gain further insights into the processes occurring in the BV2 cells upon LPS and POS treatment, analyses of GO, KEGG, Reactome, and WikiPathways enrichments were performed. The analysis revealed that DEGs between the LPS group and control group were significantly enriched in “type II interferon signaling (IFNG)” as well as inflammatory pathways including “inflammatory response”, “cellular response to interleukin-1”, “positive regulation of interleukin-6 production”, “TNF signaling pathway”, “NF-kappa B signaling pathway”, and “IL-17 signaling pathways” (**Supplementary Figure 1A**). Furthermore, the highest enrichment for the inflammatory signaling pathways outlined above was observed among the upregulated DEGs in LPS-challenged microglia (**Supplementary Figure 1B**). Next, we investigated the effects of LPS on the gene expression profiles of downregulated DEGs in BV2 cells through enrichment analysis. The results indicated a pronounced enrichment in several biological processes, including those related to the “lysosomal lumen”, “Lysosome”, “P2Y receptors”, “negative regulation of mitochondrial fission”, “Tyrobp causal network in microglia”, “regulation of protein metabolic process”, “carbohydrate metabolic process”, “Metabolism of carbohydrates”, and “the negative regulation of triglyceride metabolic process” (**Supplementary Figure 1C**). Our findings provide further evidence that LPS treatment induces the upregulation of a range of genes associated with inflammation and immune activation in microglia, a response that is accompanied by the downregulation of catabolic processes, regardless of treatment duration or cell type (27).

**Figure 2:**
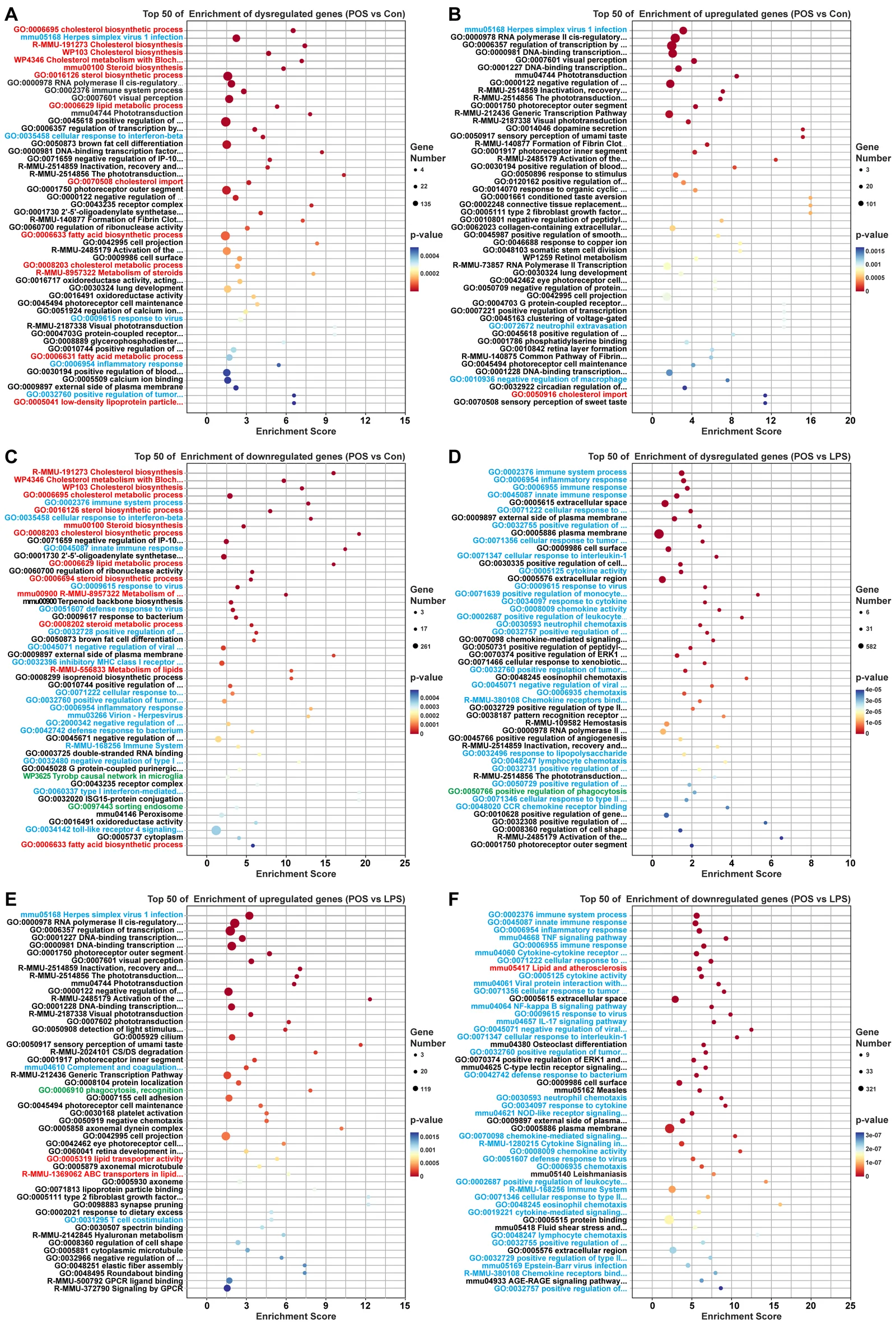
RNA sequencing analysis reveals transcriptomic pathway alterations in POS-treated BV2 cells compared with the control and LPS groups. **(A–C)** The top 50 statistically enriched terms derived from GO, KEGG, Reactome, and WikiPathways associated with DEGs, upregulated DEGs, and downregulated DEGs in the POS-treated group when compared to the untreated control group. **(D–F)** The top 50 statistically enriched terms derived from GO, KEGG, Reactome, and WikiPathways associated with DEGs, upregulated DEGs, and downregulated DEGs in the POS-treated group when compared to the LPS-treated group. Red: enriched terms relative to lipid metabolism; Cyan: enriched terms relative to immune response; Green: enriched terms relative to phagocytosis.

Unlike the LPS treatment, the analysis of DEGs following the POS challenge, compared with the control group, revealed significant enrichment in lipid metabolism pathways. These pathways included the “cholesterol biosynthetic process”, “Cholesterol biosynthesis”, “Cholesterol metabolism with Bloch and Kandutsch-Russell pathways”, “Steroid biosynthesis”, “sterol biosynthetic process”, “lipid metabolic process”, “cholesterol import”, “fatty acid biosynthetic process”, and “fatty acid metabolic process”. Furthermore, there was significant enrichment in immune-related pathways, including “Herpes simplex virus 1 infection”, “immune system process”, “cellular response to interferon-beta”, and “response to virus” (**Figure 2A**). Further analysis revealed that the aforementioned lipid metabolism pathways were enriched among downregulated DEGs in the POS-treated group compared with the untreated group (**Figure 2C**). The enrichment analysis of downregulated DEGs in microglia challenged with POS revealed several immune pathways, including “immune system process”, “cellular response to interferon-beta”, “negative regulation of IP-10 production”, “innate immune response”, “response to virus”, “defense response to virus”, “positive regulation of interferon-beta production”, “cellular response to lipopolysaccharide”, “positive regulation of tumor necrosis factor production”, “inflammatory response”, “type I interferon-mediated signaling pathway”, and “toll-like receptor 4 signaling pathway” (**Figure 2C**). When the POS-treated group was compared with the LPS-treated group, immune pathways associated with inflammation and interferon responses, as well as lipid metabolism pathways, were predominantly enriched among the downregulated DEGs (**Figures 2D–F**). Collectively, these results strongly suggest that POS exposure induced a unique transcriptional signature in microglia, which was markedly different from the LPS-induced signature.

### The POS challenge triggered reprogramming of lipid metabolism in microglia *in vitro*

**Figure 3:**
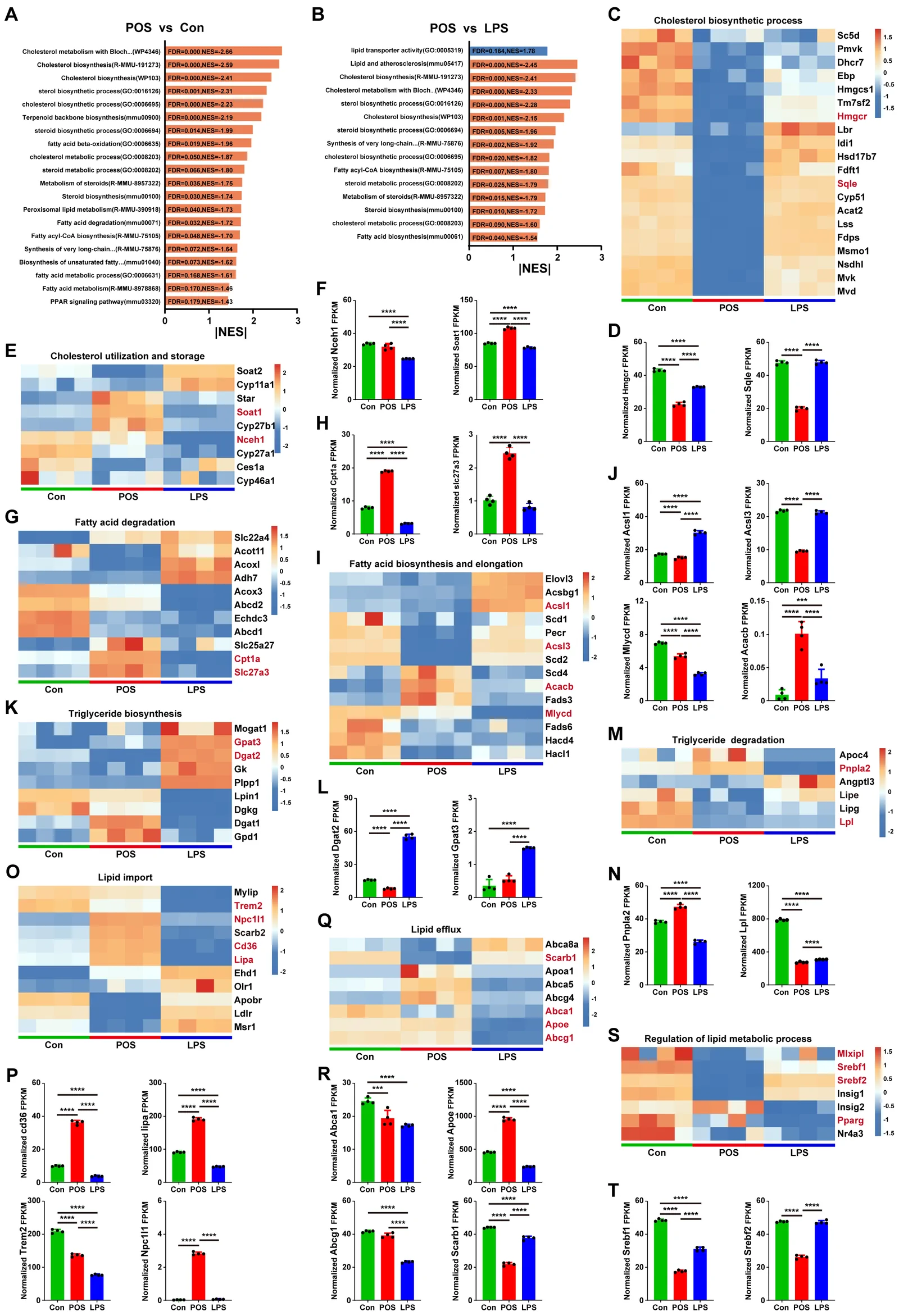
The stimulation of POSs and LPS changes the transcriptomic signature of lipid metabolism in BV2 cells. **(A)** Gene set enrichment analysis (GSEA) of DEGs shows enriched lipid metabolism pathways in the POS versus the control group. **(B)** Gene set enrichment analysis (GSEA) of DEGs shows enriched lipid metabolism pathways in the POS versus the LPS group. **(C)** Hierarchically clustered heatmaps illustrate gene expression associated with the cholesterol biosynthetic process in BV2 cells. Color key corresponds to the row Z-score; key genes are indicated by red. **(D)** Normalized FPKM of Hmgcr and Sqle in BV2 cells. **(E)** Hierarchically clustered heatmaps illustrate gene expression associated with the cholesterol utilization and storage in BV2 cells. Color key corresponds to the row Z-score; key genes are indicated by red. **(F)** Normalized FPKM of Soat and Nceh1 in BV2 cells. **(G)** Hierarchically clustered heatmaps illustrate gene expression associated with fatty acid degradation in BV2 cells. Color key corresponds to the row Z-score; key genes are indicated by red. **(H)** Normalized FPKM of Cpt1a and Slc27a3 in BV2 cells. **(I)** Hierarchically clustered heatmaps illustrate gene expression associated with the fatty acid biosynthesis and elongation in BV2 cells. Color key corresponds to the row Z-score; key genes are indicated by red. **(J)** Normalized FPKM of Alscl1, Alscl3, Acab, and Mylcd in BV2 cells. **(K)** Hierarchically clustered heatmaps illustrate gene expression associated with the triglyceride biosynthesis in BV2 cells. Color key corresponds to the row Z-score; key genes are indicated by red. **(L)** Normalized FPKM of Dgat2 and Gpat3 in BV2 cells. **(M)** Hierarchically clustered heatmaps illustrate gene expression associated with triglyceride degradation in BV2 cells. Color key corresponds to the row Z-score; key genes are indicated by red. **(N)** Normalized FPKM of Pnpla2 and Lpl in BV2 cells. **(O)** Hierarchically clustered heatmaps illustrate gene expression associated with lipid import in BV2 cells. Color key corresponds to the row Z-score; key genes are indicated by red. **(P)** Normalized FPKM of Cd36, Trem2, Lipa, and Npc1l1 in BV2 cells. **(Q)** Hierarchically clustered heatmaps illustrating gene expression associated with lipid efflux in BV2 cells. Color key corresponds to the row Z-score; key genes are indicated by red. **(R)** Normalized FPKM of Abca1, Apoe, Abcg1, and Scarb1 in BV2 cells. **(S)** Hierarchically clustered heatmaps illustrate gene expression associated with the regulation of lipid metabolic process *in vitro*-treated BV2 cells. Color key corresponds to the row Z-score; key genes are indicated in red. **(T)** Normalized FPKM of Srebf1 and Srebf2 in BV2 cells. Bars represent means; error bars represent SDs. For all comparisons, p-value was determined by Wald–test with multiple-testing correction by DESEQ2; ***p < 0.001, ****p < 0.0001.

**Figure 4:**
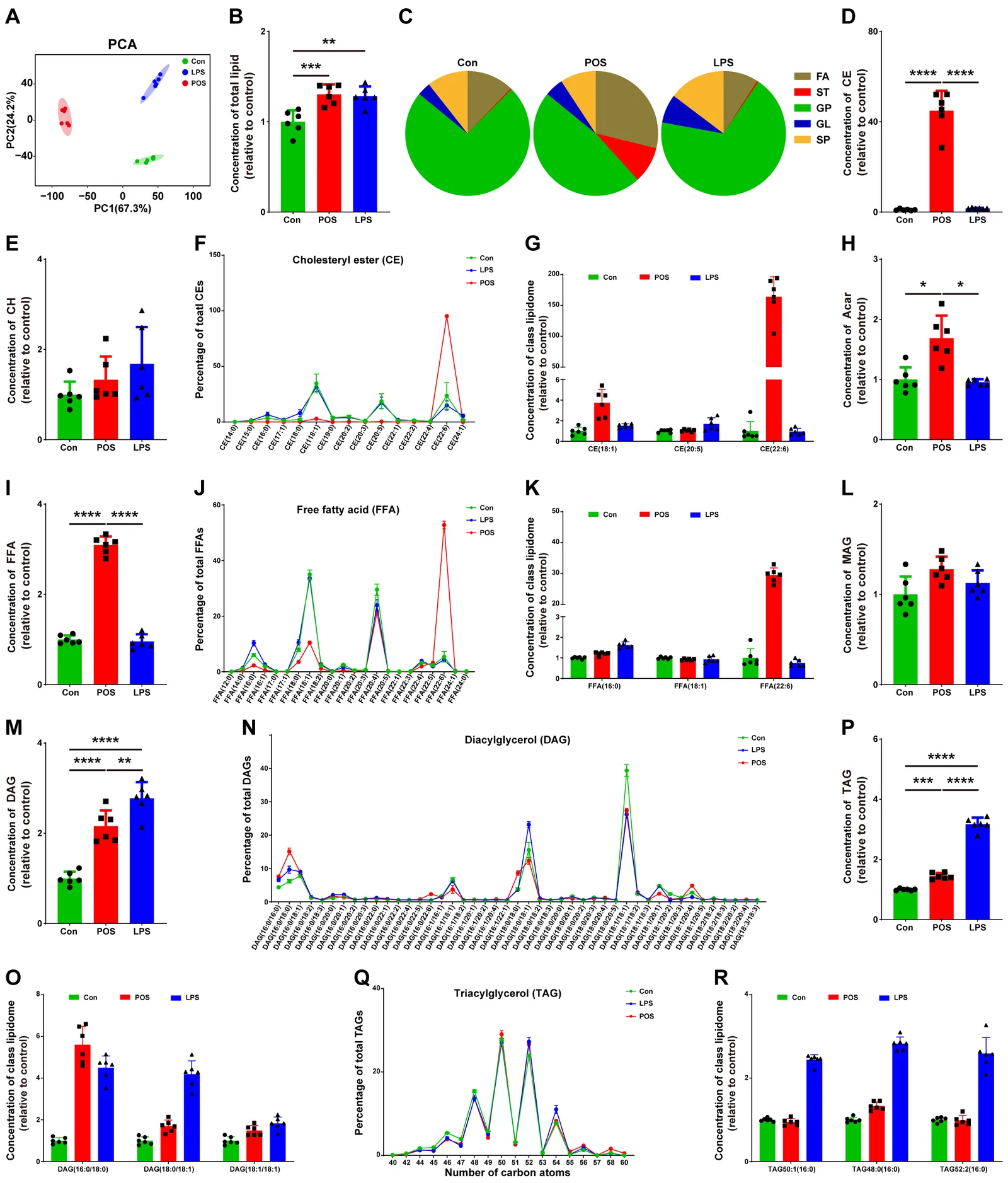
Stimulation with POSs and LPS profoundly changes the microglial lipidome *in vitro*. **(A)** PCA plot of lipidomic samples: control (green), LPS (blue), and POS (red) (n = 6 samples per group). **(B)** Overall composition of lipid metabolites in BV2 cells from three groups detected by lipidome profiling. **(C)** Total lipid concentration is normalized to the control group. **(D, E)** The concentration of total Acars and FFAs is normalized to the control group. **(F)** The distribution of FFA species in total FFAs. **(G)** The concentration of FA (16:0), FA (18:0), and FA (22:0) is normalized to the control group. **(H, I)** The concentration of CH and total CEs is normalized to the control group. **(J)** The distribution of CE species in total CEs. **(K)** The concentration of CE (18:1), CE (20:5), and CE (22:6) is normalized to the control group. **(L, M)** The concentration of total MAGs and DAGs is normalized to the control group. **(N)** The distribution of DAG species in total DAGs. **(O)** The concentration of DAG (16:0/18:0), DAG (18:0/18:1), and DAG (18:1/18:1) is normalized to the control group. **(P)** The concentration of total TAGs is normalized to the control group. **(Q)** The distribution of TAG species in total TAGs. **(R)** The concentration of TAG 50:1(16:0), TAG 48:1(16:0), and TAG 52:1(16:0) is normalized to the control group. PCA, principal component analysis; Acar, acylcarnitine; FFA, free fatty acid; CH, cholesterol; CE, cholesteryl ester; MAG, monoacylglycerol; DAG, diacylglycerol; TAG, triacylglycerol. Bars represent means; error bars represent SDs. *p < 0.05; **p < 0.01, ***p < 0.001, ****p < 0.0001 using one-way ANOVA test and Tukey multiple comparisons test **(C, D, E, H, I, L, M, P)**.

As lipid metabolism pathways were significantly enriched, we proceeded with further transcriptomic analysis to elucidate the cellular reprogramming of lipid metabolism in response to POS. Specifically, GSEA of statistically significant dysregulated genes between LPS-treated and control groups demonstrated that LPS treatment upregulated the “Triacylglyceride synthesis” pathway and downregulated the “fatty acid beta-oxidation”, “Mitochondrial long chain fatty acid beta-oxidation”, and “ABC transporters” pathways (**Supplementary Figure 1D**). Compared to the LPS-treated and untreated groups, GSEA showed that POS markedly downregulated some lipid metabolism pathways. In particular, pathways such as “Cholesterol biosynthesis”, “steroid biosynthetic process”, “fatty acid beta-oxidation”, “Fatty acid degradation”, and “fatty acid metabolic process” were significantly affected (**Figures 3A, B**). Next, we performed lipidomic analysis on BV2 cells from three groups. 686 common lipid species across all samples were detected, which were categorized into 28 distinct lipid classes (**Supplementary Figures 2A–C**). The distinct lipid profiles of microglia in each group were analyzed using principal component analysis (PCA) (**Figure 4A**). The POS and LPS groups showed similar total lipid concentrations, both significantly higher than the control group (**Figure 4B**; **Supplementary Dataset 4**). Following normalization for total lipid content, treatment with POS significantly increased the levels of fatty acyls (FAs) and sterol lipids (STs); conversely, treatment with LPS significantly increased the levels of glycerolipids (GLs) (**Figure 4C**; **Supplementary Dataset 4**). Therefore, our data indicated a distinct lipidomic transition occurring within microglia with exposure to POS and LPS.

**Figure 5:**
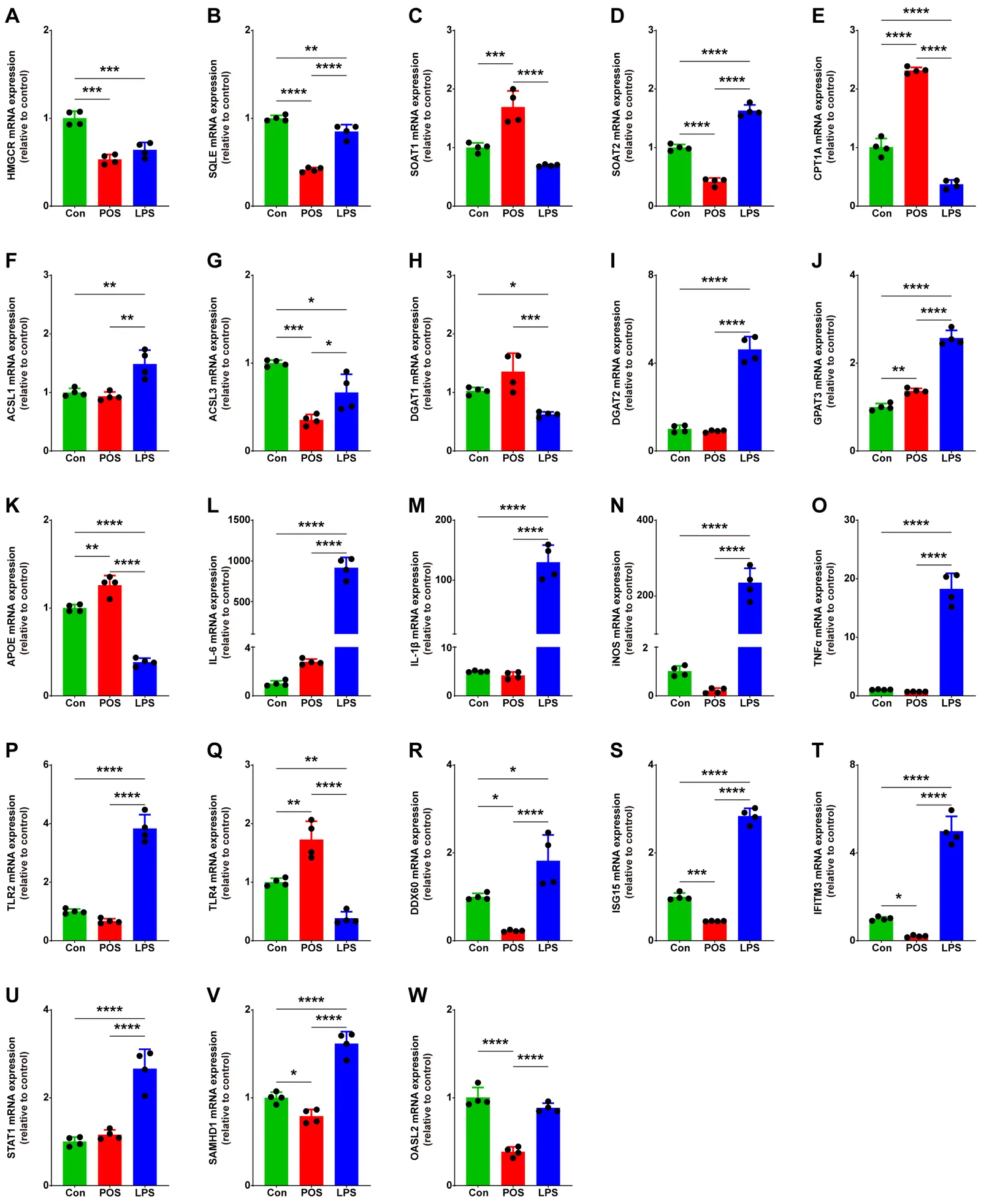
The effects of POSs and LPS treatment on the expression of key genes associated with lipid metabolism, inflammatory signaling, and interferon response in BV2 cells. **(A–K)** RT-qPCR analysis shows relative mRNA levels of lipid metabolism genes in BV2 cells across the control, LPS-treated, and POS-treated groups (n = 4 samples per group). **(L–Q)** RT-qPCR analysis shows relative mRNA levels of important inflammatory signaling genes in BV2 cells across the control, LPS-treated, and POS-treated groups (n = 4 samples per group). **(R–W)** RT-qPCR analysis shows relative mRNA levels of interferon response genes in BV2 cells across the control, LPS-treated, and POS-treated groups (n = 4 samples per group). Bars represent means; error bars represent SDs. *p < 0.05; **p < 0.01, ***p < 0.001, ****p < 0.0001 using one-way ANOVA test and Tukey multiple comparisons test **(A-V)**.

Since the signaling pathways related to cholesterol metabolism showed the most significant differences between the POS and control groups, we next explored the effect of POS on microglial cholesterol metabolism. Bulk RNA-seq across three microglial groups showed that POS exposure markedly inhibited the expression of genes involved in cholesterol biosynthesis (**Figure 3C**). Expression levels of Hmgcr and Sqle, which encode rate-limiting enzymes in the cholesterol biosynthetic pathway, were decreased in the POS group (**Figures 3D**, **5A, B**), indicating that POS exposure inhibited microglial cholesterol biosynthesis. Subsequently, the process of cholesteryl utilization and storage was examined, given that macrophages are unable to degrade sterols (28). The analysis demonstrated that treatment with POS and LPS dynamically regulated cholesteryl ester-metabolizing enzymes (**Figure 3E**). Soat1 and Soat2 encode the enzymes responsible for synthesizing cholesteryl esters (CEs) from free cholesterol. Only Soat1 was significantly upregulated following POS treatment (**Figures 3E, F**, **5C, D**). In contrast, Nceh1 and Ces1a, which encode enzymes involved in CE hydrolysis, did not exhibit significant upregulation in the POS group (**Figures 3E, F**), suggesting that POS increased CE formation potential. Notably, genes involved in the oxysterol synthesis pathway—which includes the conversion of cholesterol to oxysterols via Cyp46a1 and Cyp27a1—were markedly downregulated in the POS and LPS groups (**Figure 3E**). Consistent with the results of transcriptomic analysis, CEs were the most differentially regulated lipid class (**Figure 4D**), with very long-chain CE (22:6) being the most upregulated lipid at 48 h of POS exposure (**Figures 4F, G**). Nonetheless, no significant changes in free cholesterol (CH) levels were observed among the three groups (**Figure 4E**), indicating that excess cholesterol was likely converted into CEs for storage in BV2 cells treated with POS.

In addition to STs, FAs represented the second most differentially regulated lipid class in microglia after 48 h of POS treatment. Analysis of genes associated with FA metabolism revealed a consistent upregulation of Slc25a27, Cpt1a, Slc22a4, and Slc27a3 in the POS group (**Figure 3G**), which promote acylcarnitine (Acar) generation, a key step in the transport of fatty acids into mitochondria for β-oxidation. CPT1, located in the outer mitochondrial membrane, is widely recognized as the primary rate-limiting enzyme in the process of fatty acid oxidation (FAO), whose primary function is to catalyze the formation of Acars (29). POS treatment significantly upregulated Cpt1a expression, whereas LPS exerted an opposing effect on Cpt1a expression (**Figures 3H**, **5E**). Acar levels increased significantly following POS treatment, although Acars constituted less than 1‰ of the FAs (**Figure 4H**). The acyl-CoA synthetase long-chain (ACSL) family of enzymes is responsible for the conversion of free fatty acids (FFAs) to fatty acyl-CoA, playing an integral role in FFA metabolism by facilitating FFA uptake for β-oxidation, triglyceride synthesis, and phospholipid biosynthesis (30). The expression levels of Acsl1, contributing to microglial triglyceride synthesis and inflammatory responses (31), exhibited a significant increase in the LPS group, whereas the expression of Acsl3 demonstrated a notable decrease in the POS group (**Figures 3I, J**, **5F, G**). FFAs derived from LPS-treated and control BV2 cells showed similar composition and levels (**Figures 4I, J**). However, upon POS exposure, FFAs became the lipid class differentially upregulated (**Figure 4I**). Specifically, very long-chain unsaturated FFAs-C22:6, also known as docosahexaenoic acid (DHA), demonstrated a significant increase, whereas the proportion of FFAs-C18:1, known as oleic acid, experienced a decline (**Figures 4J, K**). Lipid ontology (LION) enrichment analysis was employed to systematically characterize alterations in the lipidome from both biochemical and biophysical perspectives (**Supplementary Figures 2D–F**). “C22:6” was significantly enriched in the POS group compared with the LPS and control groups (**Supplementary Figures 2E, F**), indicating that DHA played a vital role in the remodeling of lipid metabolism in BV2 cells exposed to POS treatment.

Given that the biosynthesis of GLs, especially triglycerides, is essential for LPS-induced microglial activation (32), the present study focused on the GL metabolism pathway. LION enrichment analysis indicated that “glycerolipids” was the most significant pathway when comparing the LPS-treated and POS-treated groups to the untreated group (**Supplementary Figures 2D, E**). Transcriptomic analyses further identified the upregulation of several genes involved in triglyceride biosynthesis, including Mogat1, Gpat3, Dgat2, GK, and Plpp1 (**Figure 3K**). Dgat2 and Gpat3, which encode key enzymes that regulate the rate-limiting step in triglyceride biosynthesis (33), demonstrated upregulation in response to LPS exposure; conversely, treatment with POS led to a modest decrease in Dgat2 expression and a slight increase in Dgat1 expression (**Figures 3K, L**, **5H–J**). Notably, genes associated with triglyceride degradation, including Lipg, Apoc4, Lpl, and Pnpla2, were downregulated in the LPS-treated group (**Figures 3M, N**). Pnpla2 encodes the enzyme ATGL, which catalyzes the rate-limiting step of triglyceride lipolysis (34). The LPS treatment significantly decreased Pnpla2 expression in BV2 cells, while the POS treatment significantly increased Pnpla2 expression (**Figures 3M, N**). No significant differences in monoacylglycerol (MAG) levels were detected among the three groups of BV2 cells (**Figure 4L**). Consistent with previous studies (24, 32), LPS significantly increased the levels of diacylglycerols (DAGs) and triacylglycerols (TAGs), whereas the increases in DAGs and TAGs induced by POS were inconsistent (**Figures 4M, P**). Furthermore, the distribution of DAGs varied among the groups (**Figures 4N, O**). In contrast, BV2 cells from all three groups showed a similar chain-length distribution of TAG-associated fatty acids, with peaks at 48:0, 50:1, and 52:2 (**Figures 4Q, R**).

The POS treatment primarily upregulated genes associated with cholesterol uptake, including Cd36, Npc1l1, and Scarb2. In contrast, LPS treatment predominantly upregulated genes involved in lipoprotein import, such as Ldlr, Olr1, and Msr1 (**Figures 3O, P**). Lysosomal acid lipase (LAL), encoded by the lipase A (Lipa) gene, hydrolyzes CE and TGA in the lysosome to release FFAs and free cholesterol (35). Notably, Lipa gene expression in BV2 cells treated with POS increased significantly, whereas a marked decrease was observed in the LPS-treated group (**Figures 3O, P**). Abnormal lipid accumulation in macrophages induces a cellular defense mechanism that mitigates the harmful effects of excessive lipid uptake by enhancing lipid efflux (28). The LPS treatment significantly reduced the expression of lipid efflux genes in BV2 cells, whereas the POS treatment markedly upregulated their expression, including Abca5, Apoa1, Abcg4, and Apoe (**Figures 3Q, R**). Apolipoprotein E (ApoE), which plays a significant role in lipid efflux primarily through its interaction with ABCA1, is essential for microglial activation and phenotypic transformation (36). Its expression was markedly upregulated in the POS-treated group, while slightly downregulated in the LPS-treated group (**Figures 3Q, R**, **5K**). Sterol regulatory-element binding proteins (SREBPs) are transcription factors that regulate genes involved in cellular lipid metabolism and homeostasis. Specifically, SREBP1 primarily mediates global lipid synthesis and cellular growth, while SREBP2 regulates cholesterol homeostasis (37). A significant downregulation of Srebpf1 (SREBP1) and Srebpf2 (SREBP2) was observed in BV2 cells treated with POS compared to those treated with LPS and the control group (**Figures 3S, T**). Peroxisome proliferator-activated receptor gamma (PPARγ) is a lipid-activated nuclear transcription factor that regulates glucose and lipid metabolism and serves as a potent modulator with anti-inflammatory effects (38). LPS significantly inhibited Pparg (PPARγ) expression in BV2 cells (**Figure 3S**). The findings indicate that exposure to POS induces lipid metabolic reprogramming in microglia via mechanisms distinct from those observed following LPS exposure.

### The accumulation of lipid droplets was induced in POS-treated microglia *in vitro* and in degenerative retinal microglia *in vivo*

**Figure 6:**
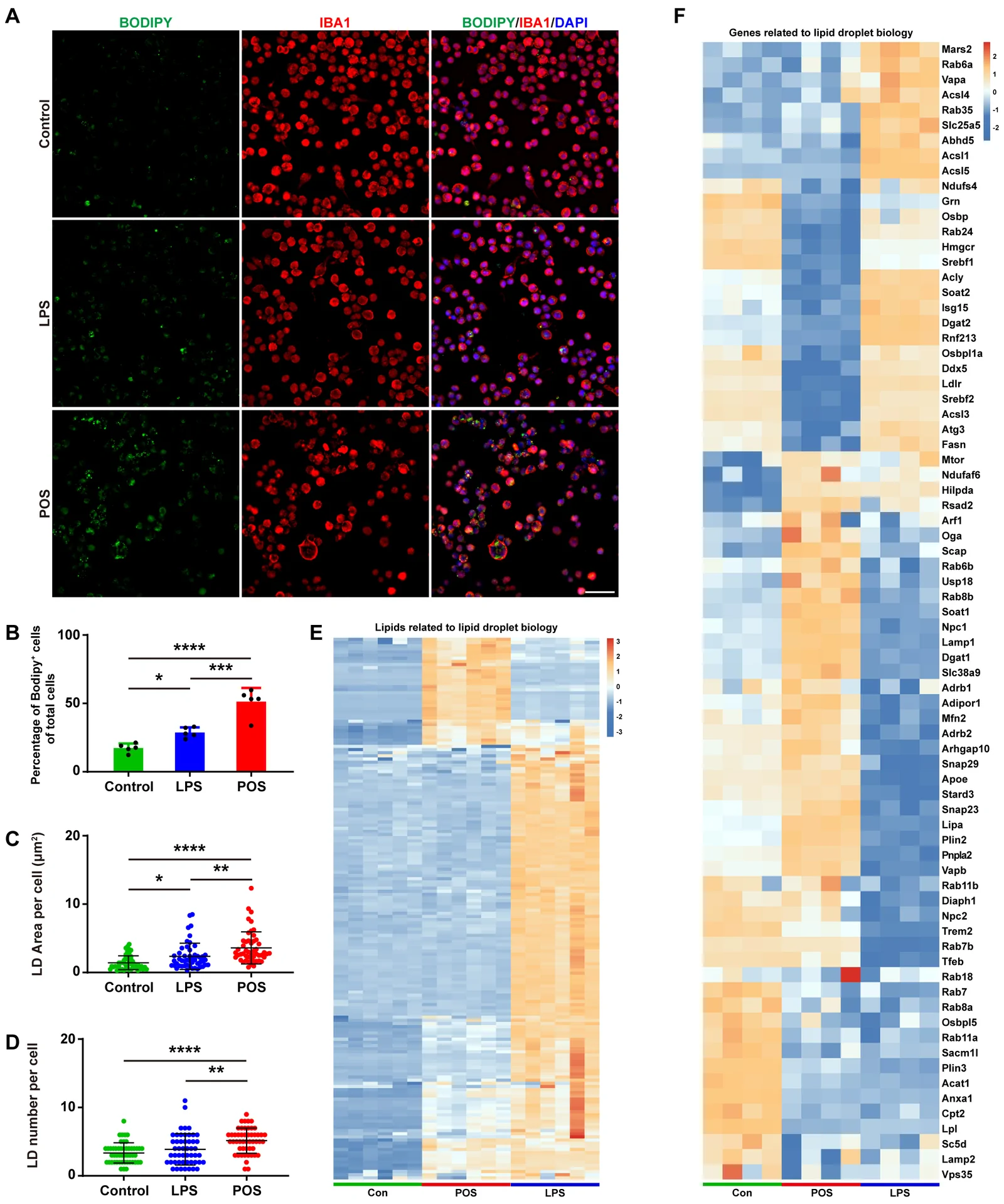
Exposure to POS and LPS induces lipid droplet accumulation in BV2 cells. **(A)** Representative high-resolution confocal images show immunofluorescent staining of BODIPY, IBA1, and DAPI in the BV2 cells across the control, LPS-treated, and POS-treated groups. Scale bar, 50 μm. **(B)** Quantification of the percentage of BODIPY^+^ cells in the BV2 cells (the number of BODIPY^+^IBA1^+^ cells/total number of IBA1^+^ cells) across the control, LPS-treated, and POS-treated groups. Bars represent means; error bars represent SDs. *p < 0.05, ***p < 0.001, ****p < 0.0001 using one-way ANOVA test and Tukey multiple comparisons test. **(C, D)** The size and number of LDs (BODIPY^+^) per cell in the control, POS-treated, and LPS-treated groups. Bars represent means; error bars represent SDs. *p < 0.05, **p < 0.01, ****p < 0.0001 using Kruskal–Wallis test and Dunn’s multiple comparisons test. **(E)** Hierarchically clustered heatmaps depict lipids related to the lipid droplet biology *in vitro*-treated BV2 cells. Color key corresponds to row Z-score. **(F)** Hierarchically clustered heatmaps depict gene expression changes of the lipid droplet biology *in vitro*-treated BV2 cells. Color key corresponds to row Z-score.

Previous studies have associated the accumulation of lipid droplets (LDs) in microglia with inflammation, aging, and neurodegenerative diseases (39). To investigate whether exposure to POS induced LD formation in BV2 cells, BODIPY, a dye that specifically labels neutral lipids, was used to examine the spatial distribution of LDs in microglia (**Figure 6A**). The percentage of BODIPY-positive cells significantly increased in both the POS- and LPS-treated groups compared with the control group (**Figure 6B**). Moreover, treatment with POS demonstrated a greater capacity for LD accumulation in BV2 cells than LPS, resulting in increased LD quantity and size per cell (**Figures 6C, D**). LION enrichment analysis identified the “Lipid droplet” pathway as significantly enriched in the POS and LPS groups compared to the control group (**Supplementary Figures 2D, E**). Specifically, the lipid metabolites associated with “lipid droplets,” which were enriched in the LION analysis, showed distinct compositions across the three BV2 cell groups, implying that LDs from each group might exhibit a distinct lipid profile (**Figure 6E**). A previously identified gene set related to lipid droplet biology was examined using transcriptomic analysis (**Figure 6F**), suggesting that POS and LPS each activate distinct molecular pathways to regulate lipid droplet biogenesis.

**Figure 7:**
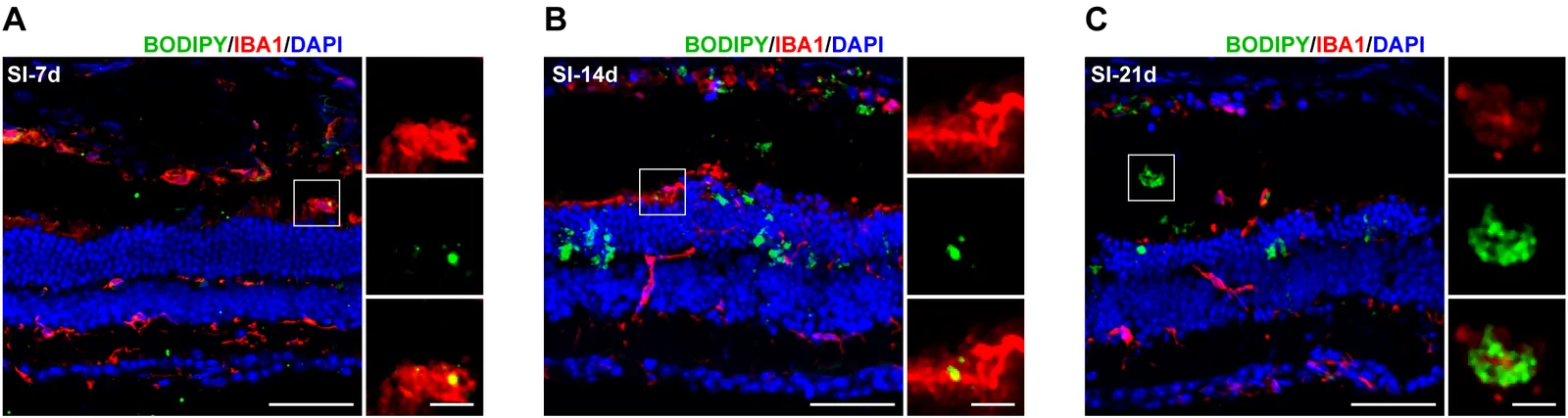
LDAM is activated in the photoreceptor layer following SI-induced retinal degeneration. **(A–C)** Representative high-resolution confocal images showing immunofluorescent staining of BODIPY, IBA1, and DAPI in the retinas of mice subjected to SI at 7, 14, and 21 days. The right panel presenting the typical expression of BODIPY in the IBA1^+^ cells in the retinas of SI-treated mice at a higher magnification. Scale bar, 50 μm or 10 μm.

To confirm whether LD formation was also induced in the degenerative retinal microglia, we employed an RD model induced by SI injection, which leads to local RPE loss followed by photoreceptor degeneration. Retinal staining for IBA1 and BODIPY demonstrated that the IBA1^+^ cells rapidly appeared in the SRS at 7 days post-injury, and maintained a large number at 14 and 21 days (**Figures 7A–C**). Moreover, a small number of IBA1^+^ cells in the photoreceptor degenerative region at 7 days post-injury exhibited significant BODIPY positive signal expression, indicating that LDs were induced in retina microglia (**Figure 7A**). The lipid-droplet-accumulating microglia (LDAM) was also observed in the photoreceptor layer at 14 and 21 days post-injury (**Figures 7B, C**). Even a set of LDAM characterized by abundant lipid droplets and decreased IBA1 expression, indicative of a foamy microglial phenotype, was observed in the SRS at 21 days post-injury (**Figure 7C**). Homeostatic microglia primarily resided in the outer and inner plexiform layers (OPL and IPL), and no LDAM was observed in the normal retina of control mice (**Supplementary Figure 3A**). Collectively, these results demonstrate the direct effect of POS exposure in promoting LD formation in microglia *in vitro*, a microglia state that was also induced in degenerative retina *in vivo*.

### POS stimulation suppressed the interferon response in BV2 microglia

**Figure 8:**
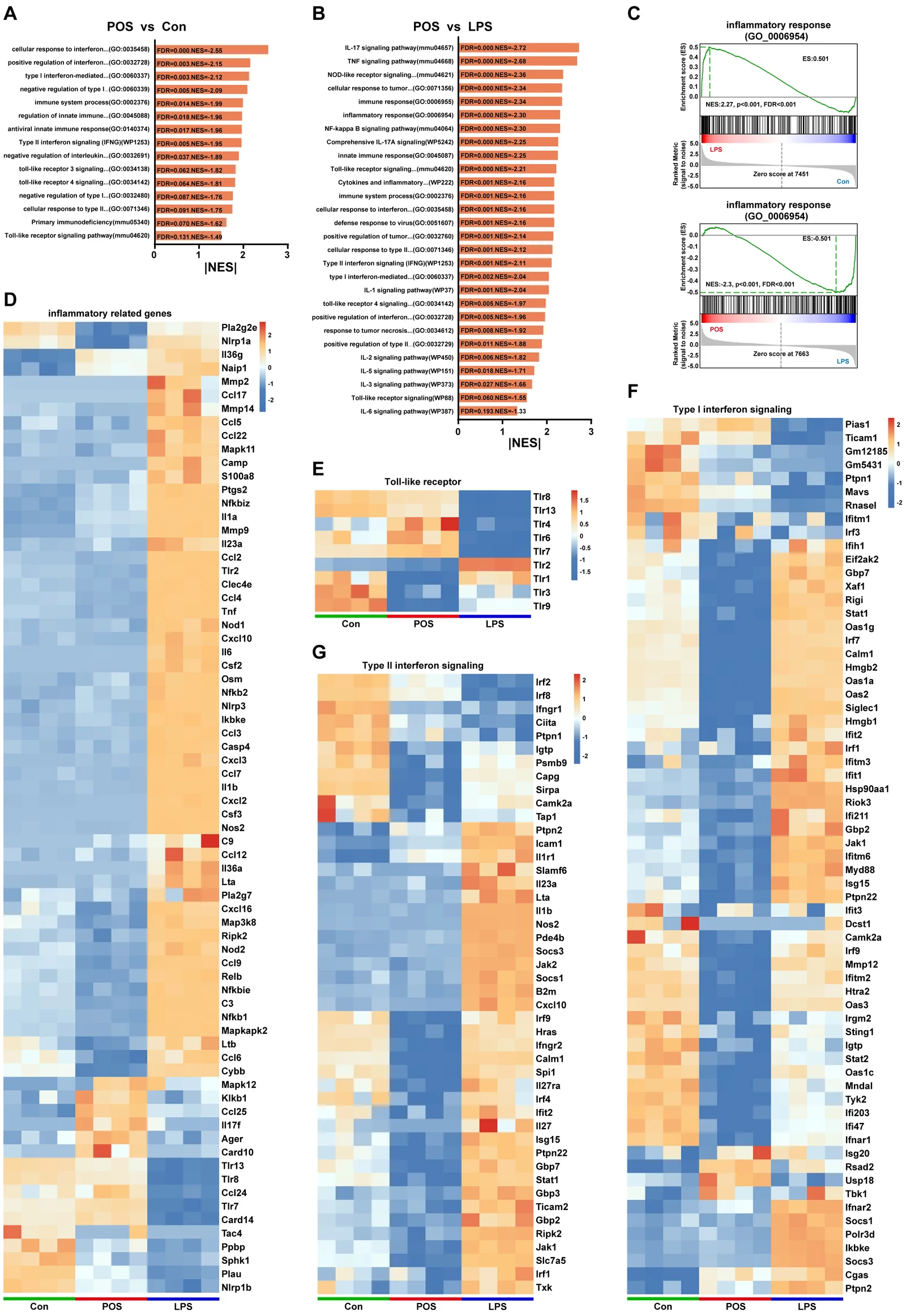
POS exposure suppresses the interferon signal pathway in BV2 cells. **(A)** GSEA of DEGs identifies the statistically significant enriched pathways associated with the immune signal pathway in the POS-treated group compared with the untreated group. **(B)** GSEA of DEGs identifies statistically significant enriched pathways associated with the immune signal pathway in the POS-treated group compared to the LPS-treated group. **(C)** GSEA identifies significantly downregulated gene sets related to the inflammatory signaling pathway in the POS-treated group compared to those in the LPS-treated group. **(D)** Hierarchically clustered heatmaps show expression changes in inflammatory genes in BV2 cells. Color key corresponds to row Z-score. **(E)** Hierarchically clustered heatmaps illustrate expression changes in toll-like receptor (TLR) genes in BV2 cells. Color key corresponds to row Z-score. **(F)** Hierarchically clustered heatmaps illustrate gene expression changes in the type I interferon signal pathway in BV2 cells. Color key corresponds to row Z-score. **(G)** Hierarchically clustered heatmaps illustrate gene expression changes in the type II interferon signal pathway in BV2 cells. Color key corresponds to row Z-score.

Another significant characteristic of LPS-treated microglia is increased immune activation, particularly involving inflammatory and interferon signaling pathways (27, 40), as confirmed by our enrichment analysis (**Supplementary Figures 1A–C**). GSEA was applied to assess the effect of POS on inflammatory and interferon signaling in BV2 cells. The results demonstrated that POS treatment significantly inhibited interferon signaling pathways, including “cellular response to interferon-beta”, “positive regulation of interferon-beta production”, “type I interferon-mediated signaling pathway”, “Type II interferon signaling (IFNG)”, and “cellular response to type II interferon”, compared with the untreated group (**Figure 8A**). In addition to the interferon signaling pathways described above, GSEA analysis comparing the POS- and LPS- treated groups further supported the downregulation of inflammatory pathways, such as “IL-17 signaling pathway”, “TNF signaling pathway”, “NF-kappa B signaling pathway”, and “Toll-like receptor signaling pathway” (**Figure 8B**). Differential gene expression analysis revealed that most inflammatory genes were upregulated in microglia from LPS-treated mice compared to untreated controls (**Figures 8C, D**). In contrast, POS treatment resulted in significant changes in the expression of only a limited subset of genes associated with the inflammatory pathway, compared with the control group (**Figure 8D**). Furthermore, RT-qPCR results revealed marked upregulation of key inflammatory genes (including IL-1β, IL-6, iNOS, and TNFα) in the LPS-treated group, without significant alterations in the POS-treated group (**Figures 5L–O**). Previous research has demonstrated that photoreceptor proteins induce microglial inflammatory responses by enhancing Toll-like receptor (TLR) signaling pathways (41). Notably, an upregulation of TLR4 was observed in BV2 cells treated with POS, whereas an upregulation of TLR2 occurred in BV2 cells treated with LPS, in comparison to the control group (**Figures 8E**, **5P, Q**). Bulk RNA-seq analysis demonstrated a significant reduction in the expression of genes associated with type I and type II interferon signaling pathways (**Figures 8F, G**). This conclusion was further supported by RT-qPCR results indicating that POSs significantly inhibited the mRNA expression of genes associated with the interferon response, such as DDX60, IFITM3, SAMHD1, OASL2, STAT1, and ISG15 (**Figures 5R–W**). It suggested that POS may exert anti-inflammatory effects and suppress interferon-responsive transcriptional programs in microglia, potentially through POS-induced reprogramming of lipid metabolism.

### Exposure of BV2 microglia to POS impaired their phagocytic ability

**Figure 9:**
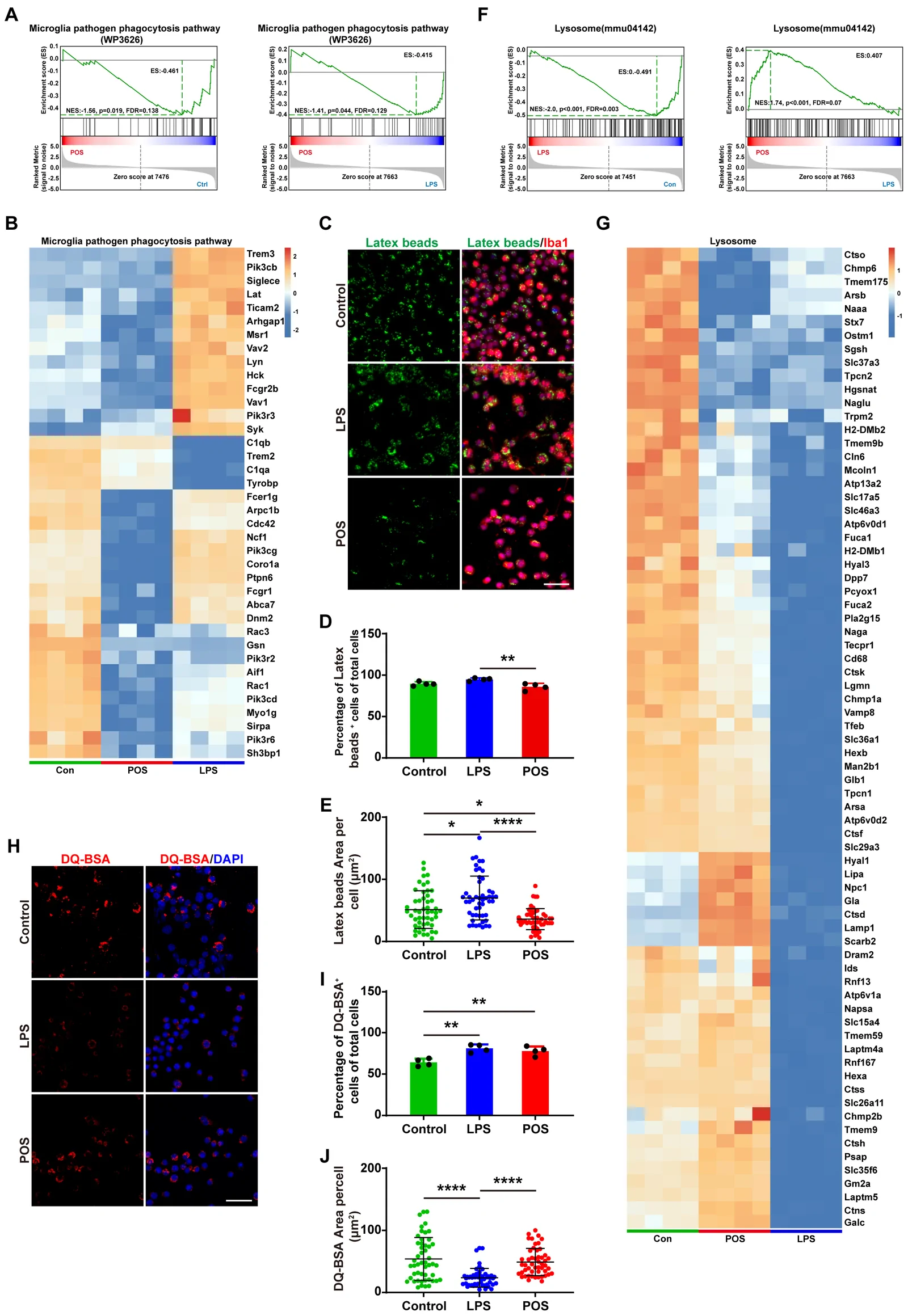
Exposure of microglia to POS results in phagocytosis deficits *in vitro*. **(A)** GSEA identifies significantly downregulated gene sets related to the microglia pathogen phagocytosis pathway in the POS-treated group compared to those in the LPS-treated group or the untreated group. **(B)** Hierarchically clustered heatmaps illustrate gene expression changes in the microglia pathogen phagocytosis pathway in BV2 cells. Color key corresponds to row Z-score. **(C)** Representative high-resolution confocal images show immunofluorescent staining of latex beads, IBA1, and DAPI in the BV2 cells from control, LPS-treated, and POS-treated groups. **(D)** Statistical analysis of the percentage of latex-beads^+^ BV2 cells (the number of latex-bead^+^IBA1^+^ cells/total number of IBA1^+^ cells) in control, LPS-treated, and POS-treated groups (n =5 samples from different groups). **(E)** Statistical analysis of the latex-bead area in each latex-bead^+^ BV2 cell of control, LPS-treated, and POS-treated groups (n = 50 cells from different groups). **(F)** GSEA identifies significantly downregulated gene sets related to the lysosome pathway in the LPS-treated group compared to those in the POS-treated or the untreated groups. **(G)** Hierarchically clustered heatmaps illustrate gene expression changes in the lysosome pathway *in vitro*-treated BV2 cells. Color key corresponds to row Z-score. **(H)** Representative high-resolution confocal images show immunofluorescent staining of DQ-BSA and DAPI in the BV2 cells from control, LPS-treated, and POS-treated groups. **(I)** Statistical analysis of the percentage of DQ-BSA^+^ BV2 cells (the number of DAPI-positive nuclei surrounded by DQ-BSA signal/total number of DAPI-positive nuclei) in control, LPS-treated, and POS-treated groups (n =5 samples from different groups). **(J)** Statistical analysis of the DQ-BSA area in each DQ-BSA^+^ BV2 cell from control, LPS-treated, and POS-treated groups (n = 50 cells from different groups). Bars represent means; error bars represent SDs. *p < 0.05; **p < 0.01, ***p < 0.001, ****p < 0.0001 using one-way ANOVA test and Tukey multiple comparisons test **(D, I)** or Kruskal-Wallis test and Dunn’s multiple comparisons test **(E, J)**.

Phagocytic activity plays an important role in microglia-mediated suppression of photoreceptor degeneration during retinal degeneration (13). Since impaired phagocytosis is a hallmark of LDAM (24), we investigated how exposure to POS affects microglial phagocytosis. Interestingly, the treatment of POSs downregulated the microglial phagocytosis of pathogens pathway, according to GSEA (**Figure 9A**). Further analysis revealed a downregulation of most microglia phagocytosis genes (**Figure 9B**). To assess whether POS-treated microglia exhibited altered engulfment capacity, BV2 cells were incubated with 1-μm fluorescent latex beads, which function as indicators of cellular engulfment (**Figure 9C**). Consistent with previous findings, LPS increased engulfment (24) (**Figures 9D, E**). Conversely, POS significantly decreased the percentage of phagocytic cells and the number of engulfed events per cell (**Figures 9D, E**), indicating that POS exposure induced deficits in engulfment of BV2 cells.

Lysosomal function is essential for sustained phagocytic activity and efficient degradation of substrate cargo (42). GSEA demonstrated that LPS-treated BV2 cells exhibited downregulation of the lysosomal pathway (**Figure 9F**). Most genes in this pathway were expressed at low levels in LPS-treated microglia, whereas minimal changes in these genes were observed between the POS-treated and control groups (**Figure 9G**). DQ-BSA is a fluorogenic substrate for proteases, whose fluorescence is quenched by labeling with BODIPY dyes, and hydrolysis of DQ-BSA into single peptides by proteases relieves this quenching (43). Therefore, the DQ-BSA assay was performed in BV2 cells to assess lysosomal function, with the fluorescent signal serving as a proxy for proteolytic activity (**Figure 9H**). Although both the POS and LPS treatments slightly increased the percentage of DQ-BSA^+^ cells (**Figure 9I**), a significant reduction in the DQ-BSA fluorescence area was found in LPS-treated microglia compared with both the POS-treated and untreated groups (**Figure 9J**), indicating impaired lysosomal degradative capacity. A modest but not significant decrease in DQ-BSA fluorescence area was observed in microglia treated with POS compared with controls (**Figure 9J**), suggesting that LDAM induced by LPS or POS exhibits phagocytic deficits attributed to different underlying mechanisms.

## Discussion

**Figure 10:**
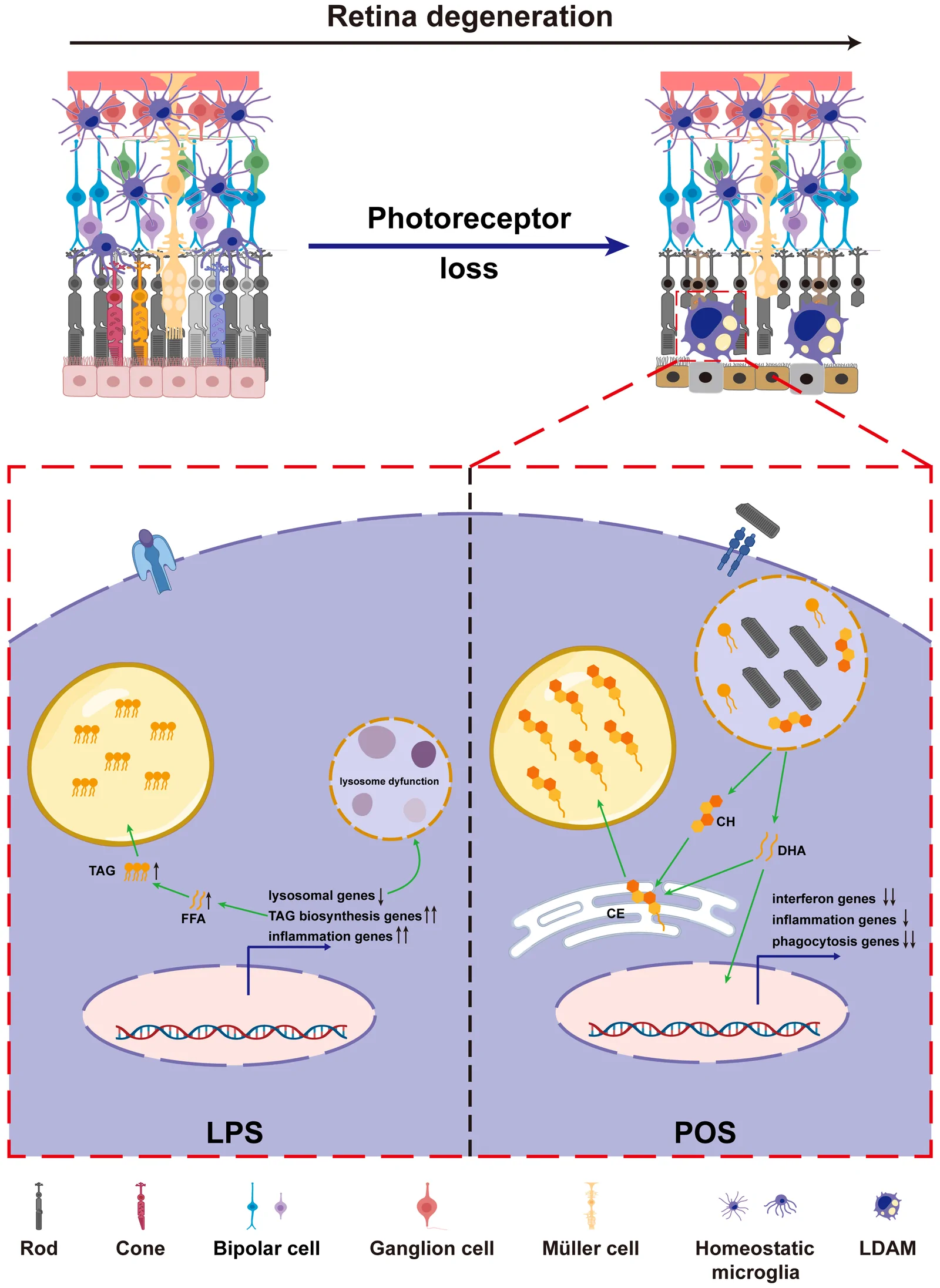
POS exposure induces LDAM activation by reprogramming microglial lipid metabolism in a manner distinct from LPS treatment.

In conclusion, this study highlights lipid metabolic reprogramming induced by POS as a key driver of microglia activation. We found that POS exposure induced a novel microglial state defined by distinct morphological features, unique transcriptional signatures, and specific lipidomic profiles, all of which differed from those observed in the LPS group. POS exposure induced reprogramming of microglial lipid metabolism, characterized by a significant increase in DHA and CEs, likely contributing to LD accumulation. Consistent with this, LDs were observed in microglia within the photoreceptor layer of the SI-induced degenerative retina, confirming the LDAM activation *in vivo*. Functionally, POS-treated microglia showed anti-immune effects and impaired engulfment compared with LPS-induced microglia. While lysosomal dysfunction observed in LPS-treated BV2 cells was absent in the POS-treated group (**Figure 10**). Thus, POS exposure induces lipid metabolic reprogramming, which contributes to LD accumulation in microglia, implying its potential role in driving microglial phenotypic switching during RD.

During photoreceptor degeneration, microglia rapidly infiltrate the photoreceptor layer and engulf stressed or dead photoreceptors (13, 19). Consequently, photoreceptor debris, including POSs, appears to play a critical role in microglial activation. The colocalization of IBA1 and rhodopsin observed in POS-treated BV2 cells indicated that POSs were engulfed by BV2 cells, mirroring the process in the retina. The result of primary-cultured microglia incubated with POS without lipids demonstrates that endogenous photoreceptor proteins contribute to TLR4-mediated microglial inflammation (41). However, in the present study, inflammation in microglia was not activated upon exposure to POS, although the expression level of the Tlr4 gene increased. Approximately 50% of the dry weight of POS consists of lipids (18, 44), suggesting that lipids in POS may contribute significantly to microglia activation.

Lipid metabolism is known to play a crucial role in regulating microglial states and functions like migration and phagocytosis (45), the dysregulation of which has been linked to neuroinflammatory processes and neurodegenerative disorders (45–47). Thus, it is also important to identify the effects of specific lipids derived from photoreceptors on microglial lipid metabolism in RD, providing functional biomarkers and delineating novel molecular mechanisms underlying microglial activation. Despite this potential, few studies have investigated the impact of photoreceptor degeneration on microglial lipid metabolism. In the present study, our findings were corroborated by previous reports indicating that LPS exposure primarily increased TAG synthesis (24, 32, 34). Compared with LPS-treated and untreated groups, cholesterol metabolism pathways were most significantly affected in POS-treated microglia. Subsequent comprehensive lipidomic analysis demonstrated that CEs were the most upregulated lipids in POS-treated microglia, further confirming that POS predominantly induces reprogramming of microglial cholesterol metabolism. Appropriate cholesterol concentrations are necessary for microglial survival both *in vitro* and *in vivo* (48). However, the portion of free cholesterol that has exceeded the cell’s buffering capacities is a prerequisite for crystallization. The accumulation of cholesterol crystals can damage microglial lysosomes and trigger a severe inflammatory response by activating the inflammasome pathway (45, 49). In the present study, free cholesterol levels in BV2 cells were not altered following POS exposure compared with those in the LPS and control groups. Furthermore, bulk RNA-seq analysis revealed upregulation of Soat1 (esterification) and Apoe (efflux) genes, indicating maintenance of cellular cholesterol homeostasis in POS-treated microglia, likely due to promoting cholesterol esterification into LDs and enhancing cholesterol efflux, thereby reducing the pool of free cholesterol available for crystallization. This conclusion might be further supported by minimal alterations in lysosomes and inflammation. Genes involved in cholesterol biosynthesis were markedly downregulated, while genes related to cholesterol import were significantly upregulated in microglia exposed to POS, suggesting that excessive cholesterol accumulation may result from phagocytic clearance of cholesterol released from POS.

An excess of FFAs in their non-esterified form is considered indicative of dysregulated lipid homeostasis, which can induce cellular dysfunction and lipotoxicity (50). Microglia can eliminate excess FFAs, undergo oxidative degradation via β-oxidation in mitochondria and peroxisomes, and convert them into TAGs (45, 51). Together with findings from previous reports, no significant changes in the levels and distribution of FFAs were observed in LPS-treated microglia compared with untreated microglia, suggesting that they may be metabolized into TGs (24, 32, 45, 52). In contrast, DHA, comprising approximately 52% of total FFAs in POS-treated BV2 cells, was the most upregulated FFA compared with the LPS-treated and untreated groups. Moreover, the level of cholesterol ester DHA (CE-22:6) increased significantly in microglia exposed to POS, accounting for approximately 95% of total CEs. The significant accumulation of CE-22:6 in microglia is likely due to SOAT1-mediated esterification of free cholesterol with DHA, which underscores the critical role of DHA in regulating cholesterol homeostasis. Previous studies have demonstrated that DHA exhibits anti-oxidant and anti-inflammatory effects by preventing NF-κB translocation to the nucleus during microglial activation (53–55). A comparable effect has been reported in macrophages, where DHA inhibits LPS-induced cholesterol synthesis, proinflammatory signaling, and IFN-regulated genes (56). The majority of POS lipids are glycerophospholipids, and the remainder is largely comprised of cholesterol (~10 mol% of total lipids) and glycolipids (18, 44). Moreover, POS phospholipids are highly enriched in DHA, which constitutes over 50% of the fatty acid side chains of POS phospholipids (57). In the mouse model of inherited RD, increased LDAM numbers and decreased DHA levels have been observed in the degenerative retina. Dietary DHA supplementation enhances photoreceptor survival and converts activated microglia to a quiescent phenotype (58). It is hypothesized that exogenous DHA derived from POS plays a critical role in remodeling lipid metabolism, thereby contributing to microglial activation and phenotypic transformation.

LDs are neutral lipid-filled, with a core composed primarily of neutral lipids—mainly TAGs and CEs, enclosed by a single phospholipid monolayer (59). The accumulation of LDs in microglia, giving rise to a distinct population termed LDAM, can be triggered by various pathological stimuli, such as LPS, long-chain fatty acids, stroke, traumatic brain injury, aging, neurodegeneration, and even tauopathy neurons (39, 46, 60, 61). LDAM, induced by aging, inflammation, and neurodegeneration, has been characterized by excessive TAG accumulation, phagocytic defects, increased reactive oxygen species, and elevated proinflammatory cytokine levels (24, 31, 32, 39, 51, 62). Taken together, TAGs have been shown to be key immunomodulatory molecules in both normal and pathological microglial physiology (45, 63). Blocking LD accumulation by inhibiting TAG synthesis in LPS-stimulated microglia attenuates the inflammatory response, whereas inhibiting microglial LD lipolysis exerts beneficial effects in acute neuroinflammatory conditions (34, 52). Appropriate TAG synthesis sequesters lipids into LDs, thereby limiting their availability for participation in inflammation signaling pathways, while the lipolytic machinery of excess TAGs in LDs might provide a highly regulated, on-demand source of inflammatory lipid mediators (64, 65). It partly explained a twist of fate in the regulation of microglial function by TAGs. CEs, another major lipid class linked to LDs, accumulate in microglia after the engulfment of cholesterol-rich cell debris and further induce the foamy microglial/macrophage state (49, 66, 67). Microglia containing cholesterol-rich LDs, a key defining feature of demyelinating lesions, initially facilitate disease recovery by clearing damaged myelin. However, the chronic presence of foamy microglia/macrophages is associated with increased demyelination (39). Cholesterol crystals, rather than CEs in LDs, have been suggested to be the main drivers of chronic neuroinflammation in foamy microglia (49, 68). In the present study, microglia treated with POS did not exhibit increased free cholesterol levels, lysosomal damage, or neuroinflammation, further supporting the conclusion that the accumulation of CE-rich LDs might help inhibit cholesterol dysregulation and microglial dysfunction (49). Therefore, the effects of LDs on microglial activation depend on lipid content, which induces different LDAM subtypes with functional heterogeneity.

DAM, a conserved state associated with neurodegeneration, is characterized by a purposeful transcriptional reprogramming toward debris clearance by enhancing phagocytosis, lipid metabolism, and lysosomal activity (69). Many homeostatic and DAM genes were downregulated in LPS- and POS-treated BV2 cells compared with the untreated group. The activation of the Erk1/2 pathway by LPS has been suggested to be a key mechanism that suppresses homeostatic and DAM genes *in vivo* (70). DHA treatment significantly inhibits the expression of IRF-1 mRNA (71), a transcriptional factor that positively regulates core DAM genes (70), which particularly explains the mechanism by which POSs downregulated the expression of some DAM genes. LD accumulation has been observed in plaque-associated DAMs derived from human iPSCs in AD mice (72). Furthermore, prolonged Aβ exposure promotes microglial LD formation via the conversion of FFAs to TGs (31, 51), implying that lipid metabolic remodeling triggered by various pathological stimuli might induce the transformation of DAM to LDAM. During photoreceptor degeneration, microglia migrate into the photoreceptor layers and are activated into a DAM phenotype, contributing to the engulfment of POS (6, 9, 13, 73–75). Consistent with previous reports (58, 76), LDAM was mainly observed in the SRS in the RD model in our study. Whether POS induces LD accumulation in DAM and triggers DAM transformation to LDAM will be determined in our future studies. In our previous study, the number of IRM in the GCL increases as the disease progresses, alleviating the loss of RGSs in the retina following optic nerve crush (22). The IFN-I signaling pathway has also been suggested to be associated with microglial migration during photoreceptor degeneration. The number of IRM increases at the early stage, reaches a peak when microglia are abundantly present in SRS, then declines rapidly (7). Our results demonstrated that POS exposure significantly suppressed microglial interferon signaling, suggesting that POS exposure might inhibit IRM activation and delay photoreceptor loss.

This study has several limitations. The culture system for BV2 cells may not accurately replicate the state of microglia in the retina, as the transcriptomic characteristics of microglia *in vitro* cultures differ significantly from those observed *in vivo (*77). In addition, there are no rescue or inhibition experiments to confirm mechanistic conclusions. Although the expression level of DAM and IRM genes decreased in the BV2 cells treated with POS, the effect of POS on DAM and IRM activation in the photoreceptor layer remains unclear. A subset of LDAM was found in the SRS in an RD model, while the role of LDAM in photoreceptor loss and its detailed mechanism needs to be clarified *in vivo*. Single-cell or spatial transcriptomics could improve the understanding of the effect of POS on microglial phenotypic transformation in future studies.

In conclusion, this study has identified the effect of POS exposure on microglia activation, highlighting lipid metabolic reprogramming as a key driver of LDAM during RD. However, POS-induced LDAM exhibited different functions compared with LDAM induced by LPS, aging or other neurodegenerative diseases. The underlying mechanism might be that DHA from POSs played a key role in maintaining cholesterol homeostasis in POS-induced LDAM by facilitating the esterification of free cholesterol into CEs, thereby suppressing their detrimental function. Thus, these findings suggest that therapeutic strategies targeting microglial lipid metabolism hold significant potential to protect surviving photoreceptors by regulating microglia activation. Additionally, it is preferable to use an *in vitro* POS-treated microglial model to investigate or confirm the mechanism underlying microglial activation in degenerative retinas rather than LPS-treated models.

## Data Availability

The datasets presented in this study can be found in online repositories. The names of the repository/repositories and accession number(s) can be found in the article/**Supplementary Material**.

## Supplementary material

Supplementary Figure 1 RNA sequencing analysis reveals transcriptomic pathway alterations when comparing LPS treatment with control group in BV2 cells *in vitro*. **(A-C)** The top 50 statistically enriched terms derived from GO, KEGG, Reactome, and WikiPathways associated with DEGs, upregulated DEGs, and downregulated DEGs in the LPS-treated group when compared to the untreated control group. Red: enriched terms relative to lipid metabolism; Cyan: enriched terms relative to immune response; Green: enriched terms relative to phagocytosis. **(D)** Gene set enrichment analysis (GSEA) of DEGs shows enriched lipid metabolism pathways in the LPS versus the control group.

Supplementary Figure 2 Lipid alterations in LPS-treated and POS-treated microglia *in vitro*. **(A–C)** Classification bubble charts show fold changes (log2 scale) and significance (log10 scale) of lipids in BV2 cells across the untreated group, the LPS-treated group, and the POS-treated group (n = 6 samples from distinct groups). **(D–F)** Lipid ontology enrichment analysis in BV2 cells across the untreated group, the LPS-treated group, and the POS-treated group.

Supplementary Figure 3 LDAM is not observed in the normal retina of control mice. **(A)** Representative high-resolution confocal images showing immunofluorescent staining of BODIPY, IBA1, and DAPI in the retinas of control mice. The right panel presenting the typical expression of BODIPY and IBA1^+^ cells in the retinas of control mice at a higher magnification. Scale bar, 50 μm or 10 μm.

Supplementary Table 1 Primers used for RT-qPCR.
