# Supplementary material for: Photoreceptor outer segments promote the accumulation of lipid droplets in microglia by reprogramming lipid metabolism
Source: Front Immunol. 2026 Sep 9;17:1892763. doi: 10.3389/fimmu.2026.1892763 (PMC13597234; doi:10.3389/fimmu.2026.1892763)
Supplement: Supplementary file 1 [file DataSheet2.docx]

**Supplementary Figures**

Supplementary Figure 1. RNA sequencing analysis reveals transcriptomic pathway alterations when comparing LPS treatment with control group in BV2 cells in vitro.

A-C. The top 50 statistically enriched terms derived from GO, KEGG, Reactome, and WikiPathways associated with DEGs, upregulated DEGs, and downregulated DEGs in the LPS-treated group when compared to the untreated control group. Red: enriched terms relative to lipid metabolism; Cyan: enriched terms relative to immune response; Green: enriched terms relative to phagocytosis.

D. Gene set enrichment analysis (GSEA) of DEGs shows enriched lipid metabolism pathways in the LPS versus the control group.

Supplementary Figure 2. Lipid alterations in LPS-treated and POS-treated microglia in vitro.

A-C: Classification bubble charts show fold changes (log2 scale) and significance (log10 scale) of lipids in BV2 cells across the untreated group, the LPS-treated group, and the POS-treated group (n = 6 samples from distinct groups).

D-F: Lipid ontology enrichment analysis in BV2 cells across the untreated group, the LPS-treated group, and the POS-treated group.

Supplementary Figure 3. LDAM is not observed in the normal retina of control mice.

A. Representative high-resolution confocal images showing immunofluorescent staining of BODIPY, IBA1, and DAPI in the retinas of control mice. The right panel presenting the typical expression of BODIPY and IBA1^+^ cells in the retinas of control mice at a higher magnification. Scale bar, 50 μm or 10 μm.

**Tables**

Table S1. Primers used for RT-qPCR.

| Name | Sequence |
| --- | --- |
| mGAPDH-F | GGTTGTCTCCTGCGACTTCA |
| mGAPDH-R | TGGTCCAGGGTTTCTTACTCC |
| mDDX60-F | ACTGGAACACTCGCTTTGG |
| mDDX60-R | GAAGTAGACATCACCCAACAGG |
| mIFITM3-F | CCCCCAAACTACGAAAGAATCA |
| mIFITM3-R | ACCATCTTCCGATCCCTAGAC |
| mSAMHD1 -F | GAGAATCGTGGTTTCCGAGAG |
| mSAMHD1 -R | CTCCAAGGAACTTACTCCCAGA |
| mOASL2-F | CAAGAAGTCAGGGTGATTAAGG |
| mOASL2-R | GAACAGAATCATGTCTTGGTCA |
| mSTAT1-F | GGCGAAGAGCGACCAGAAAC |
| mSTAT1-R | AGCTGATCCAGGCAGGCATT |
| mISG15-F | GGTGTCCGTGACTAACTCCAT |
| mISG15-R | TGGAAAGGGTAAGACCGTCCT |
| mHmgcr-F | AGCTTGCCCGAATTGTATGTG |
| mHmgcr-R | TCTGTTGTGAACCATGTGACTTC |
| mSqle-F | AGTTCGCTGCCTTCTCGGATA |
| mSqle-R | GCTCCTGTTAATGTCGTTTCTGA |
| mSoat1-F | GAAGGCTCACTCATTTGTCAGA |
| mSoat1-R | GTCTCGGTAAATAAGTGTAGGCG |
| mSoat2-F | TTACACCCTGGAAAACGGAAAG |
| mSoat2-R | CATGTGGTAGATGGTTCGGAAA |
| mCpt1a-F | CTCCGCCTGAGCCATGAAG |
| mCpt1a-R | CACCAGTGATGATGCCATTCT |
| mGpat3-F | TTCGAGGAAGGGACTTCGAG |
| mGpat3-R | AGGAGGTTCCATGATACTAGCTC |
| mDgat1-F | TCCGTCCAGGGTGGTAGTG |
| mDgat1-R | TGAACAAAGAATCTTGCAGACGA |
| mDgat2-F | GCGCTACTTCCGAGACTACTT |
| mDgat2-R | GGGCCTTATGCCAGGAAACT |
| mAcsl1-F | TGCCAGAGCTGATTGACATTC |
| mAcsl1-R | GGCATACCAGAAGGTGGTGAG |
| mAcsl3-F | AACCACGTATCTTCAACACCATC |
| mAcsl3-R | AGTCCGGTTTGGAACTGACAG |
| mApoe-F | GACCCAGCAAATACGCCTG |
| mApoe-R | CATGTCTTCCACTATTGGCTCG |
| mTlr4-F | GCCTTTCAGGGAATTAAGCTCC |
| mTlr4-R | GATCAACCGATGGACGTGTAAA |
| mTlr2-F | GCAAACGCTGTTCTGCTCAG |
| mTlr2-R | AGGCGTCTCCCTCTATTGTATT |
| mIL1b-F | GAAATGCCACCTTTTGACAGTG |
| mIL1b-R | TGGATGCTCTCATCAGGACAG |
| mIL6-F | TAGTCCTTCCTACCCCAATTTCC |
| mIL6-R | TTGGTCCTTAGCCACTCCTTC |
| mTNFa-F | GGTGCCTATGTCTCAGCCTCTT |
| mTNFa-R | GCCATAGAACTGATGAGAGGGA |
| miNOS-F | GTTCTCAGCCCAACAATACAAGA |
| miNOS-R | GTGGACGGGTCGATGTCAC |
